# Externally validated deep learning model to identify prodromal Parkinson’s disease from electrocardiogram

**DOI:** 10.1038/s41598-023-38782-7

**Published:** 2023-07-29

**Authors:** Ibrahim Karabayir, Fatma Gunturkun, Liam Butler, Samuel M. Goldman, Rishikesan Kamaleswaran, Robert L. Davis, Kalea Colletta, Lokesh Chinthala, John L. Jefferies, Kathleen Bobay, G. Webster Ross, Helen Petrovitch, Kamal Masaki, Caroline M. Tanner, Oguz Akbilgic

**Affiliations:** 1 Cardiovascular Section, Department of Internal Medicine, Wake Forest School of Medicine, Medical Center Boulevard, Winston-Salem, NC 27157 USA; 2 Quantitative Sciences Unit, Department of Medicine, Stanford University, Palo Alto, CA USA; 3 Division of Occupational, Environmental, and Climate Medicine, San Francisco Veterans Affairs Medical Center, University of California-San Francisco, 4150 Clement Street, Box 127, San Francisco, CA 94121 USA; 4 Department of Biomedical Informatics, Emory University, Atlanta, GA USA; 5 Center for Biomedical Informatics, University of Tennessee Health Science Center, Memphis, USA; 6 Department of Neurology, Edward Hines Jr. VA Hospital, Hines, IL USA; 7 Department of Preventive Medicine, University of Tennessee Health Science Center, Memphis, TN USA; 8 Parkinson School of Health Sciences and Public Health, Loyola University Chicago, Maywood, IL USA; 9 Veterans Affairs Pacific Islands Health Care Systems, Honolulu, HI USA; 10 Pacific Health Research and Education Institute, Honolulu, HI USA; 11 Kuakini Medical Center, Honolulu, HI USA; 12 Department of Geriatric Medicine, John A. Burns School of Medicine, University of Hawaii, Honolulu, HI USA; 13 Department of Neurology, Weill Institute for Neurosciences, University of California San Francisco, San Francisco, CA USA

**Keywords:** Parkinson's disease, Computer science, Predictive markers

## Abstract

Little is known about electrocardiogram (ECG) markers of Parkinson’s disease (PD) during the prodromal stage. The aim of the study was to build a generalizable ECG-based fully automatic artificial intelligence (AI) model to predict PD risk during the prodromal stage, up to 5 years before disease diagnosis. This case–control study included samples from Loyola University Chicago (LUC) and University of Tennessee-Methodist Le Bonheur Healthcare (MLH). Cases and controls were matched according to specific characteristics (date, age, sex and race). Clinical data were available from May, 2014 onward at LUC and from January, 2015 onward at MLH, while the ECG data were available as early as 1990 in both institutes. PD was denoted by at least two primary diagnostic codes (ICD9 332.0; ICD10 G20) at least 30 days apart. PD incidence date was defined as the earliest of first PD diagnostic code or PD-related medication prescription. ECGs obtained at least 6 months before PD incidence date were modeled to predict a subsequent diagnosis of PD within three time windows: 6 months–1 year, 6 months–3 years, and 6 months–5 years. We applied a novel deep neural network using standard 10-s 12-lead ECGs to predict PD risk at the prodromal phase. This model was compared to multiple feature engineering-based models. Subgroup analyses for sex, race and age were also performed. Our primary prediction model was a one-dimensional convolutional neural network (1D-CNN) that was built using 131 cases and 1058 controls from MLH, and externally validated on 29 cases and 165 controls from LUC. The model was trained on 90% of the MLH data, internally validated on the remaining 10% and externally validated on LUC data. The best performing model resulted in an external validation AUC of 0.67 when predicting future PD at any time between 6 months and 5 years after the ECG. Accuracy increased when restricted to ECGs obtained within 6 months to 3 years before PD diagnosis (AUC 0.69) and was highest when predicting future PD within 6 months to 1 year (AUC 0.74). The 1D-CNN model based on raw ECG data outperformed multiple models built using more standard ECG feature engineering approaches. These results demonstrate that a predictive model developed in one cohort using only raw 10-s ECGs can effectively classify individuals with prodromal PD in an independent cohort, particularly closer to disease diagnosis. Standard ECGs may help identify individuals with prodromal PD for cost-effective population-level early detection and inclusion in disease-modifying therapeutic trials.

## Introduction

Parkinson’s disease (PD) is a systemic disease that is currently diagnosed when classic motor symptoms including resting tremors and bradykinesia, become evident and generally manifest after 50% of substantia nigra dopaminergic neurons are already dead or dying and with extensive striatal dopaminergic deafferentation^1–4^. However, pathologic changes in the lower brainstem and peripheral autonomic nervous system likely begin years or even decades before significant nigral damage occurs^5,6^, and symptoms referable to these sites of early pathologic injury may manifest many years before motor PD is diagnosed^3^. Putative interventions to delay or slow the progression from prodromal disease to parkinsonism could be implemented during this pathologic evolution if PD patients could be identified early with confidence.

Lewy pathology is found throughout the autonomic nervous system in PD^7^. Pathology of the sympathetic and parasympathetic ganglia, cardiac nerves and cardiac deafferentation are consistently seen in early PD^8–11^ and post-mortem examinations associate these pathologies with *incidental* nigral Lewy bodies (ILB)^10^. For this reason, cardiac sympathetic deafferentation as measured by ^123^I-Metaiodobenzylguanidine (^123^I-MIBG) scintigraphy is designated as a supportive criterion for the clinical diagnosis of PD in the MDS-PD diagnostic criteria^12^. However, MIBG scintigraphy is invasive and expensive, and is not a viable tool for population-level screening for PD risk.

Sympathetic and parasympathetic nerve inputs mediate adaptive responses to varying physiological conditions and cardiovascular demands, all of which can manifest as heart rate variability (HRV). HRV can be assessed by measuring the duration between consecutive R waves on an ECG^13^. HRV is reduced in PD, likely reflecting underlying cardiac autonomic Lewy pathology and deafferentation^14–22^. In addition, HRV has recently been shown to correlate with striatal dopamine depletion in PD^23^. Paralleling MIBG observations, evidence suggests that HRV may be reduced in prodromal PD. HRV is reduced in rapid eye movement (REM) sleep behavior disorder (RBD)^24,25^, a condition that is thought to be a biomarker and strongest predictor for prodromal PD, due to its direct correlation to the progression to PD^26,27^.

A previous study using data from Atherosclerosis Risk in Communities Study Description (ARIC), a large prospective study, found that low HRV was associated with 2-3 fold increased subsequent risk of PD over a mean of 18 years follow up period^28^. In addition, there has been research into predicting PD or prodromal PD using numerous clinical risk factors in combination with biological components extracted from serum samples, such as cytokines and chemokines within a decision tree algorithm^29^. Research by Akbilgic et al. (2022) used cardiac electrical activity signal information from 60 subjects from the Honolulu Asia Aging Study (HAAS) within a logistic regression model following a Probabilistic Symbolic Pattern Recognition (PSPR) method to identify patients at high risk of PD but without a pre-specified time window^30^. While this research reported an average AUC of 0.835, the HAAS cohort was a small and very homogeneous data sample and not representative of the general population. While HRV metrics, typically measured using 5-min ECGs, have been used in multiple studies, there is some debate whether such classical metrics can indeed capture substantial information on the likelihood of development of PD^30^. In previous research, models were developed to predict future PD using machine learning algorithms that incorporate multiple features derived from the full waveform of standard 10-s ECGs^30^. Such research indeed provides substantial preliminary indication that ECG-based signals can be used as a predictor of PD, with the possibility of predicting PD onset at a timely manner before diagnosis.

This research builds on prior artificial intelligence (AI) approaches but uses raw waveform ECGs rather than features extracted from ECGs. There is substantial utility, generalizability and simplicity of raw 12-lead ECGs, which are easily and routinely collected, to predict prodromal (and risk of) PD within a specified time window. Importantly, this novel study uses two independent study populations with available electronic ECGs obtained prior to PD onset, with one dataset used for model building and the other used for external validation.

## Results

Our initial EHR-based search identified 131 eligible PD cases and 1,058 controls from the MLH database, with a total of 428 and 3584 available ECGs, respectively. After validation of PD diagnosis by manual chart review, the LUC dataset included 29 PD cases and 165 matched controls with one ECG each. The baseline characteristics of these cohorts are summarized in Table 1.

**Table 1:** Baseline characteristics.

	MLH			LUC		
	Total (n = 1189)	Case (n = 131)	Control (n = 1058)	Total (n = 194)	Case (n = 29)	Control (n = 165)
Mean age at ECG (SD)	70.1 (9.5)	72.6 (8.0)	69.8 (9.6)	71.2 (8.7)	69.8 (8.7)	71.4 (8.8)
Mean age at PD diagnosis (SD)	75.3 (7.7)	75.3 (7.9)	–	72.3 (8.0)	71.0 (8.8)	–
Mean years between ECG and PD diagnosis (SD)	2.7 (1.9)	2.7 (1.9)	–	1.7 (1.0)	1.65 (1.0)	–
Male n (%)	639 (53.7%)	77 (58.8%)	562 (53.1%)	119 (61.3%)	18 (62.1%)	101 (61.2%)
Race n (%)						
White	753 (63.3%)	86 (65.7%)	667 (63.0%)	149 (76.8%)	23 (79.3)	126 (76.4)
Black or African American	412 (34.7%)	41 (31.3%)	371 (35.1%)	22 (11.3%)	3 (10.4)	19 (11.5)
Multiple	10 (0.8%)	0 (0%)	10 (1.0%)	0 (0%)	0 (0%)	0 (0%)
Other/unknown	5 (0.4%)	2 (1.5%)	3 (0.3%)	19 (9.8%)	3 (10.4)	16 (9.7)
Asian	8 (0.7%)	2 (1.5%)	6 (0.6%)	4 (2.1%)	0 (0.0%)	4 (2.4%)
Amer. Ind/Alaska Native	1 (0.1%)	0 (0%)	1 (0.1%)	0 (0%)	0 (0%)	0 (0%)

MLH, University of Tennessee-Methodist Le Bonheur Healthcare; LUC, Loyola University, Chicago.

**Figure 1:**
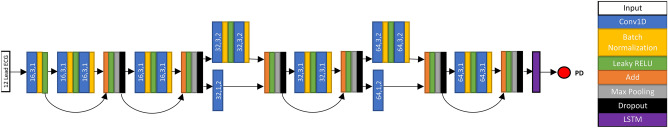
Proposed 1D-CNN architecture used to predict PD risk using 10 s 12-lead ECG data. Values in the Conv1D boxes refer to number of filters, kernel size and number of strides respectively.

**Figure 2:**
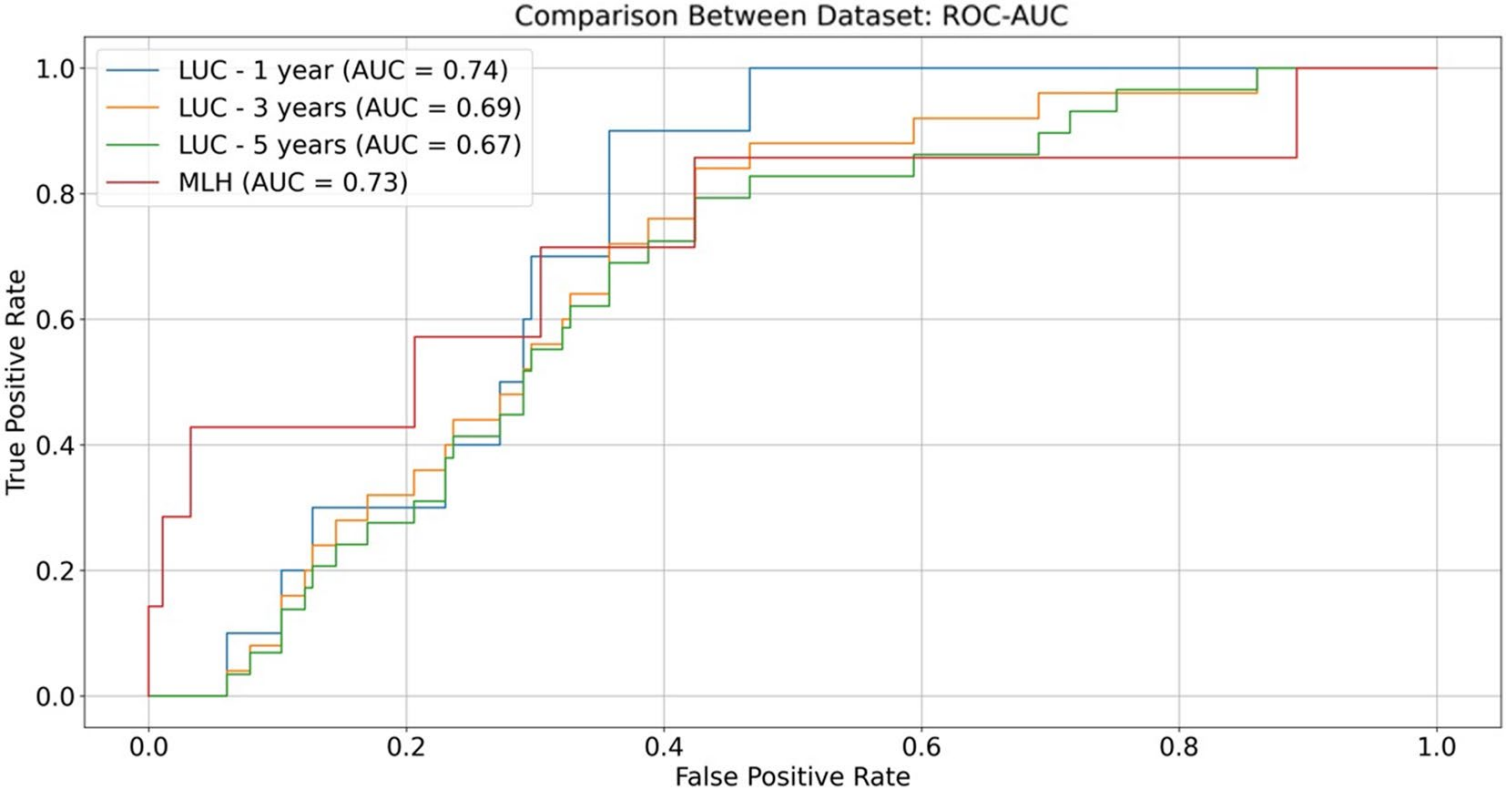
The area under the ROC Curves for LUC external validation and MLH internal validation sets.

The MLH data was split into 90% training and 10% internal validation. We developed 1D-CNN model (Fig. 1) using 90% MLH training data up to 100 epochs. The model from the epoch which resulted in the highest AUC in the 10% internal validation data was selected as the final model. This model resulted in an internal validation AUC of 0.73 with 95% CI of 0.51–0.96. To convert predicted risk values into predicted classes (PD or not), we identified the decision threshold as 0.139, which maximized the F1 score. Using this threshold, we obtained a sensitivity of 0.43 and a specificity of 0.96 in predicting prodromal PD up to 5 years before disease onset in the internal validation dataset. We note that this threshold can be adjusted according to one’s purposes, to increase sensitivity at a cost of reduced specificity. For purposes of developing a clinical trial for a putative disease-modifying drug, high specificity is the preferred metric, because it would substantially reduce the required sample size. Conversely, when one or more disease-modifying drugs become available, assuming the drug is safe, it could make sense to select a test positivity cute-point that emphasizes sensitivity over specificity, and thereby treat a larger proportion of at-risk individuals. The MLH data driven model was then externally validated on the LUC data and resulted in an AUC of 0.67 (0.54, 0.79), correctly classifying 12 of 29 PD cases 5 years prior to disease diagnosis, and 123 of 165 controls, with a specificity of 0.75 and sensitivity of 0.41. In LUC external validation data, the accuracy increased slightly when predicting future PD within 3 years of diagnosis, with AUC = 0.69 (0.57–0.82) and average precision (AP) of 0.21, with the highest accuracy achieved, as expected, when predicting PD within one year of diagnosis with an AUC of 0.74 (0.62–0.87) and AP of 0.11 (Table 2, Fig. 2).

**Table 2:** CNN model performance in the LUC external validation cohort.

Subgroup	Time from ECG until PD incident diagnosis date		
	6 months–1 year	6 months–3 years	All (6 months–5 years)
All subjects	0.74 [0.62–0.87]	0.69 [0.57–0.82]	0.67 [0.54–0.79]
Sex			
Male	0.73 [0.57–0.9]	0.71 [0.55–0.87]	0.69 [0.53–0.85]
Female	0.79 [0.6–0.98]	0.66 [0.45–0.86]	0.63 [0.43–0.84]
Race			
White	0.71 [0.56–0.86]	0.70 [0.56–0.85]	0.67 [0.52–0.81]
Black*	1.00 [1.00–1.00]	0.53 [0.13–0.91]	0.65 [0.27–1.03]
Age			
> = 60	0.73 [0.6–0.87]	0.70 [0.57–0.83]	0.67 [0.53–0.80]
< 60*	0.78 [0.40–1.15]	0.66 [0.26–1.05]	0.66 [0.26–1.05]

*The data size was very limited to reach any conclusion.

In addition to the 1D-CNN, models using multiple feature-engineering methods, including descriptive statistics extracted from 12 leads of the ECGs, sample entropy, PSPR etc., were also developed to predict prodromal PD within a 5-year time period. This was done to compare multiple models with a raw ECG-based model. From all the methods listed in Supplementary Table S1, the highest LUC validation accuracy achieved was AUC = 0.58 when using descriptive ECG features. When all the features extracted from all the different feature engineering methods were included within one main LightGBM model, the AUC increased to 0.61 (0.52–0.79). These results show that the AUC of the external validation accuracy obtained from deep learning on raw digital ECGs, which is 0.67 (0.54–0.79), was better than to those obtained from machine learning approaches using engineered ECG features (Supplementary Table S1). Since there was no major improvement in the LightGBM model utilizing all the extracted features, the 1D-CNN ECG model was the basis of the subgroup analyses.

### Subgroup analysis

Although statistical power was limited for some analyses, we implemented a comprehensive subgroup analysis of the LUC external validation cohort, stratifying by sex, race, and age. We also explored how predictive accuracy varied by time from ECG until PD incidence date (Table 2).

As shown in Table 2, prediction accuracy of the model improved with a shorter time interval between the ECG and the PD diagnosis. The 1D-CNN model predicted prodromal PD in males at the highest overall accuracy, with AUC 0.69 (0.53–0.85), although accuracy was high in women within one year of PD diagnosis (AUC 0.79 [0.60–0.98]); sex-specific differences in classification accuracy were not statistically significantly different (Delong test *p* = 0.62). Overall accuracy was similar in those aged < or > 60, though was high in those < 60 within one year of PD diagnosis (AUC 0.78 [0.40–1.15]). We had limited statistical power for race-specific comparisons.

## Discussion

There has been an increase in studies researching early identification of PD through biological models, but there still remains a major gap in early detection and, even more so, prediction of PD within the prodromal stage^31^. While multiple studies have assessed the use of various clinical features to predict prodromal PD, the implementation of such models on a population level may be limited by the lack of symptom reporting and/or diagnostic coding of these features in the medical record. Accurate identification of prodromal PD is essential for implementation of cost-effective clinical trials of putative neuroprotective agents, and ultimately for risk stratification and population-level disease prevention once therapeutic efficacy is established for one or more agents. In order to be effective on a population level, a predictive test of PD risk should be universally available, cost-effective and non-invasive. Studies found that heart rate variability (HRV) determined from 5-min ECGs is reduced in prevalent PD, and results from a single prospective study showed that lower HRV was associated with an increased risk of incident PD^32^. Prior work by our group utilized machine learning approaches to predict prodromal PD using clinical variables in one study^33^ and a proof-of-concept study using standard 10-s printed ECGs in another study^30^. Both studies resulted in moderate accuracy, however the latter study was conducted in a demographically homogeneous elderly population and lacked an external cohort for model validation^30^.

This research builds on the above work, using a deep learning approach to develop a predictive model for prodromal PD using standard 10-s raw digitized ECGs stored in a large healthcare system EHR, and validates this model using an independent cohort from a different healthcare system EHR. The developed model had moderately good classification accuracy in the validation cohort, with an AUC of 0.74 between 6 and 12 months before PD diagnosis and an AUC of 0.67 when the ECG was obtained as long as 5 years before PD diagnosis. The data imbalance between cases and controls and the relatively small size of the validation cohort likely limited our classification accuracy, yet the model successfully distinguished between the majority of cases and controls in an independent population, and has important implications as an ancillary approach to detect early PD in population-level screening.

While the AUCs derived here can be deemed as somewhat low-moderate at 5 years before diagnosis, it should be noted that the main aim of this model is to serve as a tool to help clinicians assess probable risk of people developing PD in the future and not for diagnosis per se. Other researchers have reported higher AUCs for predictive models, however most of those models were built using large numbers of extracted features including, but not limited to, clinical variables, neuropsychological test scores, domain composite scores, brain section measurements and magnetic resonance imaging (AUC = 0.84)^34,35^, while other models relied on physical movement tests (e.g. finger tapping) to extract bradykinesia features (AUC = 0.79–0.85) or PD detection based on language and/or speech data (AUC > 0.85)^36–38^. Despite some of these biomarkers are also low cost and easy to collect, they are typically collected only from already symptomatic patients, therefore, not suitable for EMR based prodromal PD screening purposes. In contrast to a standard 10-s ECG, many of these features would not be available in the EMR, limiting their usefulness for population-level screening. The use of ECGs in combination with other prodromal PD features available in the EMR will likely further improve our ability to screen the general population to identify persons at high risk for PD.

A strength of the current study is the validation of PD diagnosis and incidence dates in the external LUC validation population, thus ensuring accurate and generalizable performance metrics. Given that the model was developed in the MLH cohort without chart-based validation, a proportion of whom were likely misclassified, the predictive performance of the model in the replication cohort is all the more remarkable, and provides support for the broad generalizability of the use of ECGs for prodromal PD prediction: almost half of the future PD cases were correctly identified as such, while most of the controls were classified properly as not being at risk. We acknowledge that the deep learning model developed here needs improvement to maximize case identification, however, in addition to likely disease misclassification, it should also be noted that the MLH dataset used for training was largely imbalanced between cases and controls, making the model slightly more effective in identifying controls. The model’s performance can be improved following re-training using a larger cohort or the implementation of transfer learning in combination with a larger cohort.

In comparison to the machine learning models utilizing ECG feature-engineering as inputs (Supplementary Table S1), the CNN model predicted prodromal PD at higher accuracy. These results are comparable to published research that included multiple different biological and demographic information, some of which can be quite costly to obtain (e.g. serum analysis), within a machine learning system. Furthermore, while the prediction accuracy of the CNN model within 5 years before PD onset is moderate, this research adds to our understanding that PD cardiac markers, which are found before the motor changes of PD^39^, can act as signals within an artificial intelligence framework without the need of full ECG feature extraction. The inclusion of demographic and clinical variables as additional inputs with the predicted outcomes from the ECG CNN model will likely increase the accuracy of predicting prodromal PD within an extended time window. Karabayir et al.^33^ showed that there is added benefit in including demographic and clinical variables in the prediction of prodromal PD, allowing for opportunity for exploration of improving this work. Such variables can also extend to the inclusion of data from the Cognitive Abilities Screening Instrument (CASI), olfactory tests and the simple choice reaction times test. At the time of development of the models in the current study, such clinical and demographic variables were not available from the MLH and LUC data sources.

It is also worth noting that recent literature has showcased the potential of leveraging fractional dynamics to capture long-range memory in ECG data analysis, offering valuable insights for addressing temporal dependencies in the field^40,41^. In this study, we implemented various models including pure RNN, LSTM, and Transformers^42–44^. While RNN and LSTM models struggled to capture long-term dependencies (AUC of 0.6 and 0.61, respectively), Transformers demonstrated promise with an AUC of 0.69, effectively modeling complex temporal relationships. Nevertheless, our model, combining 1D convolution layers and LSTM, slightly outperformed Transformers by effectively capturing both short and long-term dependencies, resulting in improved accuracy. While Transformers offer comparable accuracy, their computational complexity and memory requirements may present challenges. Our model strikes a balance between efficiency and performance. In summary, our study validates the effectiveness of our hybrid model and acknowledges the promising performance of Transformers.

Overall, the CNN model developed in this research classified individuals based solely on a 10-s ECG input to an artificial intelligence framework. The easy accessibility and routine collection of ECGs within any clinical setting increases their generalizability to the wider population since it substantially reduces bias towards any sex, race or age subgroup. This makes such models useable in the wider, possibly global, population, especially with modern technological advancement which allows AI models to be incorporated within smart technologies. It is highly likely that incorporation of our ECG-based predictive model with other known prodromal markers of PD would result in improved risk stratification for PD. This highlights the importance of routine collection of ECGs within cohort studies aiming at understanding the pathophysiology of PD and other neurodegenerative diseases.

This study has some limitations. We were able to perform chart reviews on LUC data, but not on MLH data. Based on the high false positive rate of PD diagnosis based exclusively on EHR ICD codes at LUC (only 29/47 were determined to have PD), a substantial proportion of MLH PD cases were likely to have been misclassified. Therefore, implementing transfer learning on our final deep learning model and retraining it on large well-annotated datasets will result in a more accurate PD prediction tool. This also highlights the challenges around PD case ascertainment using EHR-based queries of diagnostic codes. The inclusion of AI models in clinical practice and future clinical decision support could help to reduce diagnostic misclassification. The five-year PD risk prediction model we developed correctly classified only about half of future PD cases but 96% of controls on MLH validation while only 41% of future PD cases and 75% of controls on LUC validation data. However, the test positivity cut-point can be adjusted by an investigator or clinician to emphasize high specificity (e.g., for clinical trials of putative disease-modifying drugs) or high sensitivity (e.g., for selection of high-risk individuals to receive preventive treatment), depending on the particular application.

We also note that the raw ECG data used in this study was gathered from two different cardiology information systems: GE MUSE at LUC and Epiphany at MLH. Despite this limitation, the concordant results between internal testing and the validation process provides evidence that raw ECGs exported from different platforms can be used reliably within AI frameworks, with minimal pre-processing.

The prediction accuracy of our model is highest using ECGs 6-months-1-year prior to PD diagnosis, which may pose a limitation for use in recruitment for some clinical trials. However, it still has moderate accuracy using ECGs up to 3-years or even 5-years prior to PD diagnosis. When specificity was maximized in our example (to reduce false positives), sensitivity for identifying persons with future PD was less than 50%. We acknowledge this relatively low sensitivity as a major limitation. However, for clinical trials of future disease modifying therapies, and in contrast with some diagnostic tests, high specificity is far more important than high sensitivity. We anticipate that future work that additionally incorporates demographic, clinical variables, and genetic information within the current deep learning model will offer greater sensitivity without sacrificing the high specificity of our ECG-only model. We anticipate that continuous improvement of model prediction accuracy, for example, through the use of larger and more accurate cohort data, and inclusion of other ‘simple’ variables will lead to the integration of these models within ECG-capable smart wearables. The development of smart applications within wearables will allow for cost-effective, non-invasive and non-burdensome screening of people for PD risk, allowing for early detection, quicker follow-up times and timely intervention strategies.

This work provides proof-of-principle that a deep learning predictive model using only simple 10-s ECGs correctly classifies individuals with prodromal PD with modest accuracy. This model was effective in distinguishing between future PD cases and controls in an independent cohort, increasing in accuracy closer to disease diagnosis, although still notably within the prodromal stage of the disease. The use of standard ECGs, which are easily and routinely collected by healthcare providers, may help identify individuals at high-risk of PD, allowing for timely inclusion in disease-modifying therapeutic trials and possible intervention strategies to delay or slow disease progression.

## Methods

### Standard protocol approvals and patient consents

This retrospective case–control study was conducted using data from two hospital centers in the United States (Loyola University Chicago (LUC), Maywood, IL and University of Tennessee Health Science Center (UTHSC), Memphis, TN). The study was reviewed and approved by the institutional review boards of the participating centers (Loyola University Chicago and University of Tennessee Health Science Center), and all methods were carried out in accordance with the relevant guidelines and regulations. Due to its retrospective nature and exempt status, written informed consent was waived by the institutional review boards of the participating centers.

### Study cohorts

**Figure 3:**
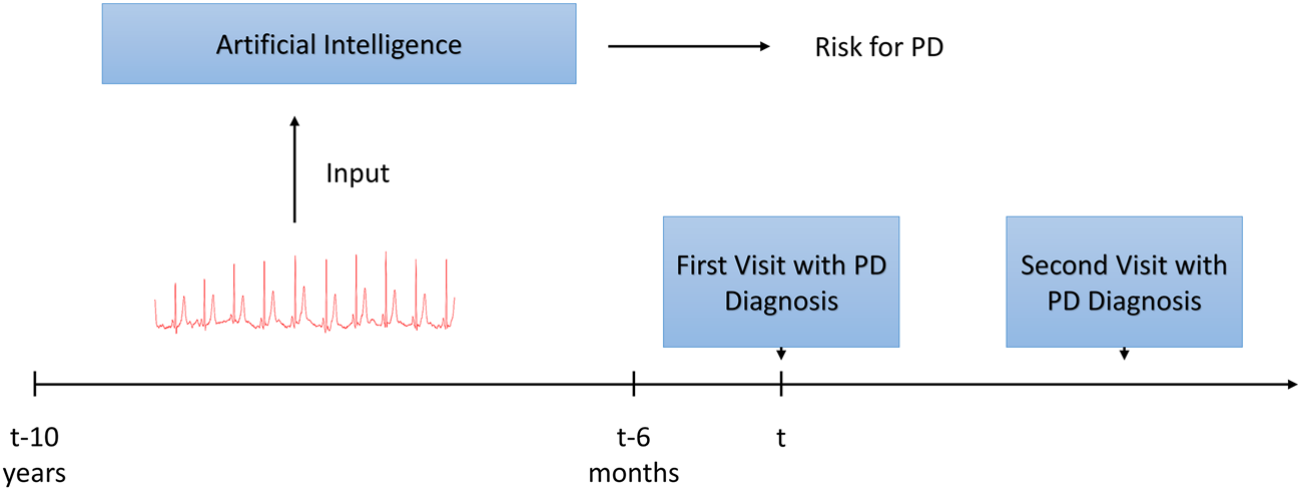
Study design.

This study used electronic health record (EHR)-based datasets from Loyola University Chicago (LUC), Chicago, IL and University of Tennessee-Methodist Le Bonheur Healthcare (MLH), Memphis, TN. The study design is summarized in Fig. 3. The participants in the analytic cohorts were aged 26–89 years at the time that the ECG was recorded. While 26 to 40 year-old participants fall within the ‘early-onset’ criteria of PD^45,46^ and is indeed rare, we utilized this information to build a more inclusive model, unbiased towards age, with the main objective of determining whether the developed models can incorporate a wide range of prodromal-to-PD scenarios.

### Case identification

In both datasets, we identified patients aged 26–89 years who had received at least two ICD diagnostic codes for PD (ICD9 332.0, ICD10 G20) as the primary reason for the visit at least 30 days apart, between May 1, 2014 and January, 2020 at LUC and January 1, 2015 and January, 2020 at MLH. PD incidence date was defined as the earliest of the first PD diagnostic code or PD-related medication prescription. For the external validation phase, we included individuals from LUC who had a standard 12-lead ECG within 5 years before the incidence date. In order to have the largest possible training sample, we included individuals from MLH within 10-years prior to the PD incidence date.

### Case validation

We reviewed the medical records of individuals from the LUC dataset to confirm the PD diagnosis and date of disease incident diagnosis. To carry out this evaluation on the LUC data, the patients’ charts were accessed using the *Epic* software, and the related clinical notes were reviewed by a movement disorders specialist (co-author KC). ‘Care Everywhere’ notes in *Epic* from other healthcare systems were also examined when available. We considered diagnostic consistency, clinical features, response to medication, and possible alternative diagnoses. All patient charts were also searched for the presence of medical diagnoses or medications potentially inconsistent with a diagnosis of PD. Based on these descriptions, we identified 131 cases at MLH and 29 eligible chart-reviewed cases (47 cases before manual chart review) at LUC and extracted their corresponding ECGs from cardio servers.

### Control identification

Randomly selected control subjects from each institution were matched to cases by age at ECG, date of ECG recording, race and sex, and were required to have been in the system for at least 10 years (for MLH) or 5 years (for LUC) with no diagnostic codes for PD or another form of parkinsonism. We identified 1058 control subjects at MLH and 65 at LUC, whose ECGs were retrieved for analysis.

### Covariate data

Covariates were also obtained from the EHR including age, sex, and race.

### ECG data

Digital 12-lead, 10-s ECG, referring to time–voltage data, was obtained for each case and control. The ECGs at LUC were exported from MUSE Cardiology Information System in XML format at 500 Hz. Base 64 encoded waveform data was further decoded using the Python programming language. ECGs at MLH were exported in DICOM format and the associated waveforms were parsed out. Some MLH ECGs were recorded at 500 Hz and some at 250 Hz and therefore, to ensure comparability and based on our previous successful application on utilization of ECG via deep learning^47,48^, all ECGs at 500 Hz were down-sampled to 250 Hz by removing one of every two amplitudes from each lead. In addition, we excluded the first second of each ECG before inputting them into the model. This was done to avoid any possible noise due to the patient’s movement at the beginning of the ECG recording. It should be noted that while ECGs were obtained from different service providers, i.e. GE MUSE and Epiphany, there is no reported difference in the ECG data exported from either service apart from the storage method (DICOM: binary vs XML: human-readable)^49^.

### Method development and external validation

Because the MLH study cohort was significantly larger than the LUC cohort, we used MLH data for training and LUC data for external validation. We note that we had access to one ECG per patient from LUC. However, we could retrieve multiple ECGs per patient at MLH, and we utilized all ECGs during training. We split the MLH dataset into 90% training and 10% internal validation sets based on patients rather than ECGs, since some patients have multiple ECGs. All available ECGs were used for the 90% training dataset. However, while multiple ECGs were available, in the 10% MLH internal validation dataset only one randomly selected ECG per patient was used. This was done with the aim of achieving representative samples to that within the LUC external validation dataset. The model’s hyperparameters were tuned based on the highest accuracy when using the internal validation set. This model, i.e. the one providing the highest AUC, was used for external validation on LUC data. In addition to the AUC, the best performing model was assessed using sensitivity, specificity, positive predictive value, negative predictive value, and average precision score (AP). Although AUC is a widely used metric to evaluate the performance of classifiers, it should be carefully utilized in evaluation of severely imbalanced classification problems since it only measures how well predictions are ranked. This makes AUC optimistic for the evaluation of classification models that include imbalanced and/or small datasets. Therefore, we have also provided the area under AP and Precision-Recall (PR) curve (AUPRC) (Figure S1) as an alternative metric to effectively evaluate the proposed model by focusing on the minority class (PD).

### Deep learning method

We used a one-dimensional convolutional neural network (1D-CNN) with raw digital ECGs as inputs with the risk for PD being the determined output. The 1D-CNN, inspired from ResNet^50^, takes advantage of skipping connections between layers, which eases the optimization of the kernels in the network and therefore obtains robust and more generalizable models than those yielded by traditional or shallow networks. CNN architectures include several hyperparameters including the number of convolutional filters, the kernel dimension, and the stride parameter of convolution layers. The parameters and the overall architecture of the CNN are depicted in Fig. 1. In our architecture, we employed 15 1D convolution layers and one LSTM layer to extract abstract spatial representations and capture both long and short-term dependencies from ECG data, enabling us to effectively capture complex patterns for accurate prediction of PD risk. To optimize the model's performance, we employed the categorical cross entropy loss function, which effectively minimized the difference between predicted and true class labels. This choice significantly improved accuracy and enabled reliable predictions, especially for multi-class classification tasks like the one presented in our work. Furthermore, we utilized the leaky rectified linear units (Leaky-RELU) activation function^51^ to obtain a better representation of the ECG by activating the intermediate neurons and allowing negative inputs. After the hyperparameter tuning process, dropout layers with a rate of 0.1 were included within the CNN architecture to penalize the kernels and help prevent model overfitting^52^. The Adam optimization algorithm^53^ (beta_1 = 0.9, beta_2 = 0.999 and learning rate = 0.001) was used to optimize the kernels in the model. The batch size for data input was set to 128. We have used the grid search approach to tune architecture based on several hyperparameters including the number of convolutional filters, the kernel dimension, and the stride parameter of convolution layers. The model’s training process was stopped once reaching consecutive decrease (five times in a row) on the AUC of the internal validation dataset.

### Conventional machine learning method

To compare the proposed deep learning algorithm, we also utilized Light Gradient Boosting Machine (LightGBM) by inputting ECG features, which were extracted from a variety of signal processing algorithms^54^. In the development of the LightGBM model, the same five-fold cross validation strategy used in the development of the CNN model was used to ensure comparability. Details of the feature engineering-based machine learning model can be found in Supplementary Material.

## Supplementary Information

Supplementary Information.

## Data Availability

Requests for patient-related data not included in the article will not be considered. The code for our proposed model can be accessed at https://github.com/ikarabayir/PD_ECG_CNN_Risk.
