# Supplementary material for: Externally validated deep learning model to identify prodromal Parkinson’s disease from electrocardiogram
Source: Sci Rep. 2023 Jul 29;13:12290. doi: 10.1038/s41598-023-38782-7 (PMC10387090; doi:10.1038/s41598-023-38782-7)
Supplement: Supplementary file 1 — Supplementary Information. [file 41598_2023_38782_MOESM1_ESM.docx]

**Supplementary Online Content**

Karabayir et. al. Externally Validated Deep Learning Model to Identify Prodromal Parkinson’s Disease from Electrocardiogram

**Results.** Results for Feature Engineering-Based Machine Learning Model

**Methods.** Feature Engineering-Based Machine Learning Model

**Figure S1.** The area under Precision-Recall curves for LUC external validation and MLH internal validation sets

**Figure S2.** The calibration curves for LUC external validation and MLH internal validation sets

**Table S1.** LUC External Validation Results of Feature Engineering Methods

**Table S2.** Confusion Matrix for the CNN model on LUC external validation (1 year)

**Table S3.** Confusion Matrix for the CNN model on LUC external validation (3 year)

**Table S4.** Confusion Matrix for the CNN model on LUC external validation (5 year)

This supplementary material has been provided by the authors to give readers additional information about their work.

*‘*

**Results.** Results for Feature Engineering-Based Machine Learning Model

A total of 156 descriptive statistics were calculated from 12 leads and among these features, 14 were selected by GA. LightGBM model with these 14 features predicted PD with an AUC of 0.58. Sample entropy of each lead was extracted, and among these features, three features were selected by GA. With LightGBM, PD was predicted with an AUC of 0.53. The list of total number of extracted and selected features from each method and their model performances in the LUC external validation dataset are shown in Table 2. The AUC of the overall model built using 51 selected ECG features was 0.61 in the LUC external validation dataset.

**Methods.** Feature Engineering-Based Machine Learning Model

We explored a variety of signal processing tools to effectively extract the underlying information present in ECG recordings. We calculated several informative time and frequency domain features and used them as predictors of PD in machine learning algorithms. These methods include descriptive statistics (DStat), Fourier Transformation (FTr), Wavelet Transformation (WTr), Sample Entropy (SEnt) and Probabilistic Symbolic Pattern Recognition (PSPR). In DStat, we calculated descriptive statistics including mean, median, kurtosis, skewness, zero crossing and mean crossing. In FTr, we calculated amplitude and phases of waves at 0.1, 0.5 and 1-40 Hz. In WTr, we implemented wavelet decomposition using db5 wavelet up to five level. In SEnt, we calculated SEnt of the whole signal for series length m=2 and used 0.25 standard deviation of each lead as a tolerance window (r). In PSPR, we modeled symbolic pattern transitions in ECG recordings.

The methods above produce several features representing ECGs. To enhance the generalizability of the classification model, we implemented a genetics algorithm (GA), which is a stochastic search algorithm to obtain an optimal subset of a set of variables and removed redundant features.

Using selected features, we first built separate machine learning models by using the extracted features from each signal processing tool. Individual decision trees tend to overfit, so we implemented the sequential ensemble method light gradient boosting machine (LightGBM) to discriminate cases and controls. We utilized Bayesian hyperparameter optimization for tuning hyperparameters in the LightGBM model, which explores the parameter space systematically instead of blind search.

**Figure S1.** The area under Precision-Recall curves for LUC external validation and MLH internal validation sets

*
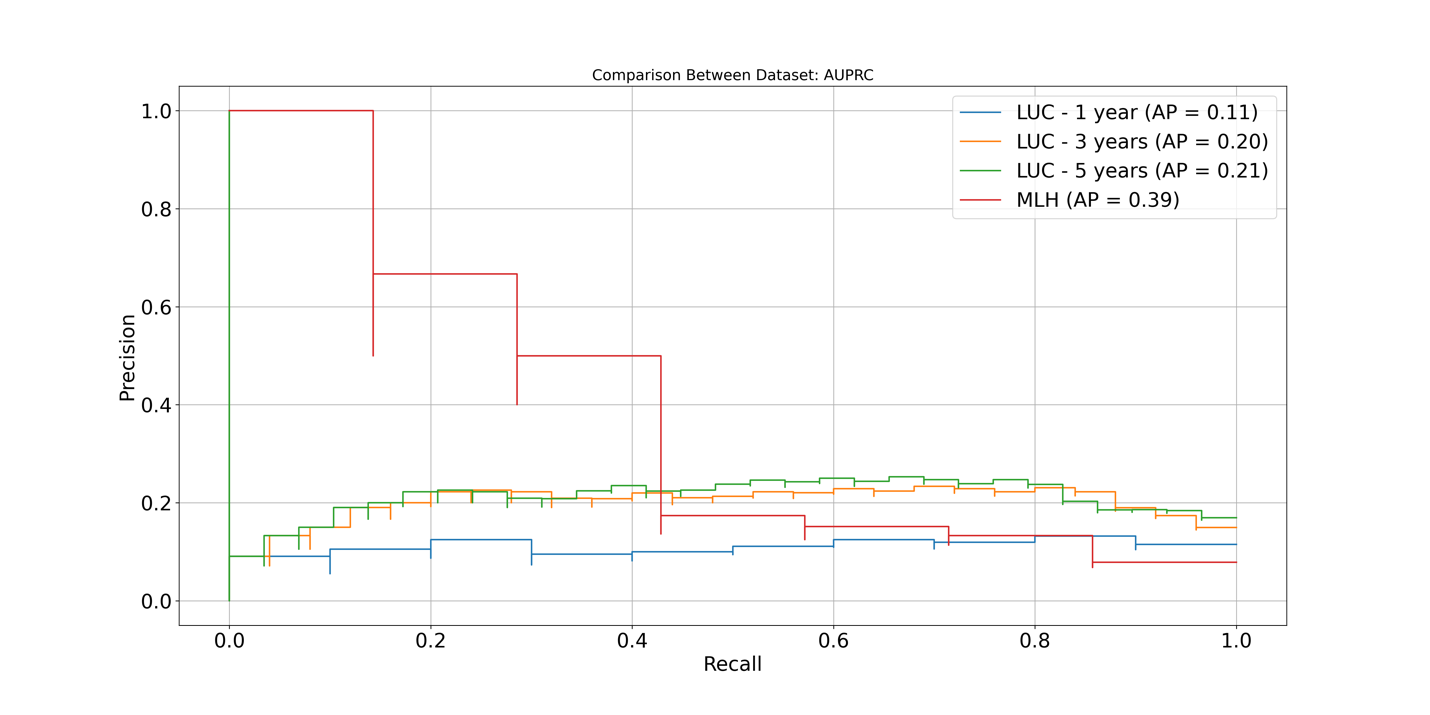
*

**Figure S2.** The calibration curves for LUC external validation and MLH internal validation sets

*
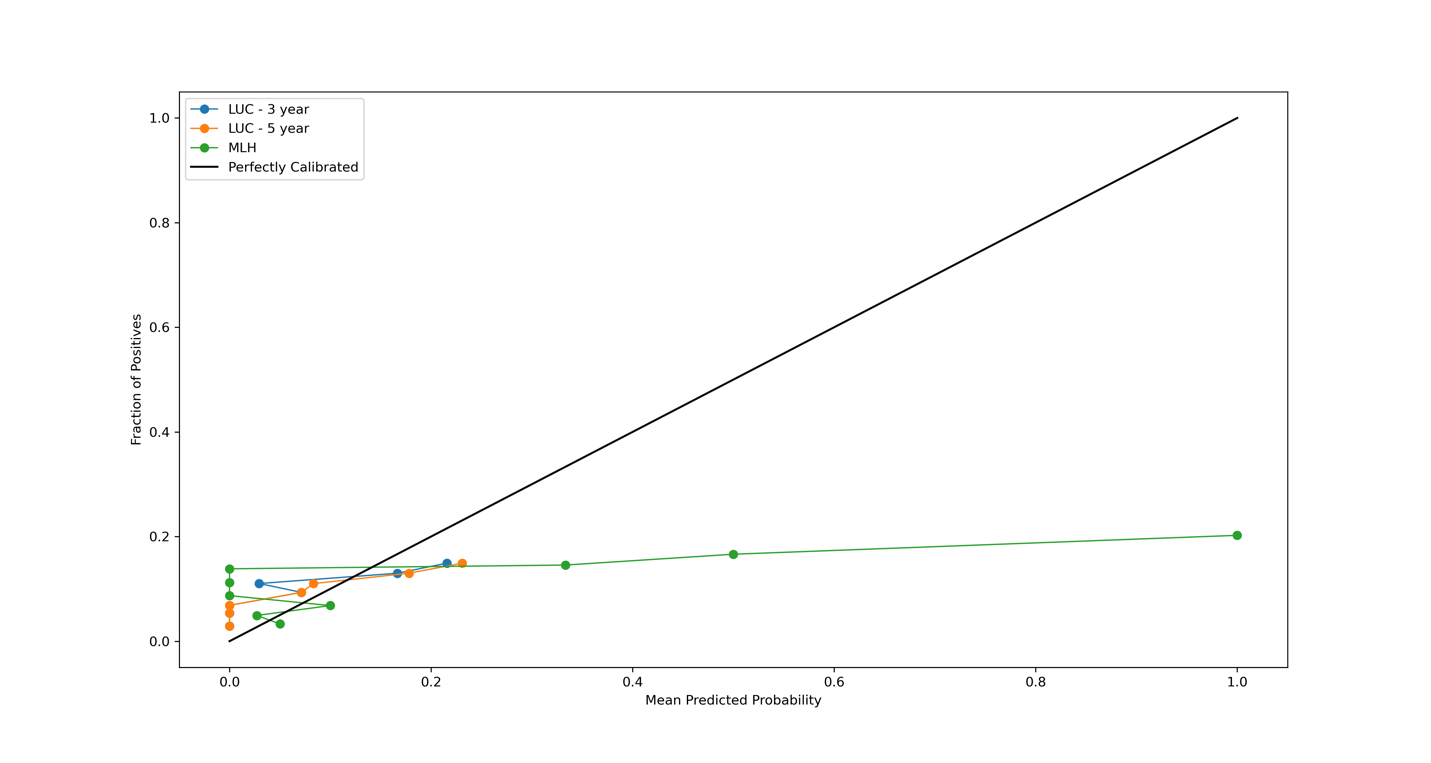
*

**Table S1.** LUC External Validation Results of Feature Engineering Methods

| **Method** | **Number of features extracted** | **Number of**  **features selected** | **AUC** |
| --- | --- | --- | --- |
| Descriptive statistics | 156 | 14 | 0.58 [0.49-0.68] |
| Sample entropy | 12 | 3 | 0.53 [0.44-0.62] |
| Probabilistic symbolic pattern recognition | 72 | 8 | 0.53 [0.43-0.62] |
| Fourier transformation | 528 | 11 | 0.54 [0.45-0.64] |
| Discrete wavelet transformation | 552 | 15 | 0.54 [0.45-0.63] |
| Total | 1320 | 51 | 0.61 [0.52-0.70] |

**Table S2.** Confusion Matrix for the CNN model on LUC external validation (1 year)

|  |  | Predicted | |
| --- | --- | --- | --- |
| Actual |  | Control | Case |
|  | Control | 123 | 42 |
|  | Case | 6 | 4 |

**Table S3.** Confusion Matrix for the CNN model on LUC external validation (3 year)

|  |  | Predicted | |
| --- | --- | --- | --- |
| Actual |  | Control | Case |
|  | Control | 123 | 42 |
|  | Case | 14 | 11 |

**Table S4.** Confusion Matrix for the CNN model on LUC external validation (5 year)

|  |  | Predicted | |
| --- | --- | --- | --- |
| Actual |  | Control | Case |
|  | Control | 123 | 42 |
|  | Case | 17 | 12 |
